# Uncovering the Neuroprotective Effect of *Hedysarum multijugum Maxim*-*Chuanxiong Rhizoma* Compound on Cerebral Infarction through Quantitative Proteomics

**DOI:** 10.1155/2022/5241902

**Published:** 2022-03-26

**Authors:** Guozuo Wang, Xiaomei Zeng, Kailin Yang, Shengqiang Gong, Anqi Ge, Wenlong Liu, Qi He, Wenhao Zhang, Jinwen Ge

**Affiliations:** ^1^Hunan University of Chinese Medicine, Changsha, Hunan Province, China; ^2^People's Hospital of Ningxiang City, Ningxiang City, Hunan Province, China; ^3^The First Affiliated Hospital of Hunan University of Chinese Medicine, Changsha, Hunan Province, China; ^4^Hunan University of Science and Technology, Xiang Tan, Hunan, China

## Abstract

**Objective:**

To uncover the neuroprotective effect of *Hedysarum multijugum Maxim*-*Chuanxiong Rhizoma* compound (Huangqi-Chuanxiong Compound (HCC)) on cerebral infarction (CI) through quantitative proteomics.

**Method:**

CI model was established by the modified Zea Longa intracavitary suture blocking method. After modeling, the rats were given intragastric administration for 7 days, once a day. After the 7-day intervention, the neurological function score was performed, the brain tissue was pathologically observed, and the total serum protein was extracted. Then, these proteins were analyzed by LC-MS/MS to identify the differentially expressed proteins (DEPs) in the HCC/CI group and CI/sham operation group. Finally, bioinformatics analysis was used to analyze DEPs, including gene ontology (GO) analysis, pathway analysis, and protein interaction analysis. ELISA and Western blotting were used to verify the proteomics results.

**Result:**

The neurological function scores of the HCC group were lower than those of the CI group. HE staining showed that the pathological results of the HCC group were improved. A total of 1340 proteins were identified by LC-MS/MS, of which 1138 proteins contain quantitative information. There are 122 DEPs in the CI/sham operation group and 25 DEPs in the HCC/CI group with fold change >1.3 or <0.77 and FDR<0.05. The 12 upregulated proteins in HCC/CI group include Protein Actn2, Kelch-like protein 41, Alpha-1, 4 glucan phosphorylase, Protein Lrtm2, Dystrophin, Galectin-1, and C4b-binding protein beta chain. The 13 downregulated proteins include Alpha-2 antiplasmin, Arachidonate 15-lipoxygenase, Carbonic anhydrase 2, Complement factor I, angiotensinogen, catalase, Protein LOC103691744, and Anionic trypsin-1. The bioinformatics analysis showed that HCC may treat CI through regulating cell-substrate adhesion and regulation, reactive oxygen species metabolic process, angiotensin response (cellular response to angiotensin), positive regulation of the occurrence of nerves and neurons (positive regulation of neurogenesis), inflammatory response, response to hypoxia (response to hypoxia, response to decreased oxygen levels), and cellular calcium homeostasis (cellular calcium ion homeostasis). The results of ELISA and Western blot also showed that, compared with model group, the angiotensinogen and catalase in HCC group were decreased (*P* < 0.05), which is consistent with the findings of proteomics.

**Conclusion:**

The therapeutic mechanism of HCC in the treatment of CI may involve fibrinolysis, cell-matrix adhesion, inflammation, hypoxia, and oxidative stress.

## 1. Introduction

Cerebral infarction (CI) is a blood supply disorders in the local brain tissue caused by various reasons, leading to necrosis of the brain tissue and resulting in corresponding neurological deficits [1]. Cerebral thrombosis is the most common type of CI, accounting for 60%–80% [2], and epidemiology showed that CI has been the second leading cause of death worldwide [2]. The main pathophysiological mechanism of CI is the complex cascade of neurons, leukocytes, and activated microglia, including excitatory amino acid toxicity, oxidative stress, inflammation, and apoptosis [1, 3, 4]. The current main therapy is tissue-type plasminogen activator (TPA), which is the only effective drug approved by the FDA for patients with acute CI [5, 6]; however, it may increase the risk of intracerebral hemorrhage [5, 6]. In addition, the delayed diagnosis and narrow treatment time window limit the application of TPA [6]. Therefore, there is an urgent need for new drugs to promote the treatment of CI.

*Hedysarum multijugum Maxim*-*Chuanxiong Rhizoma* compound (Huangqi-Chuanxiong Compound (HCC)) is a traditional Chinese medicine (TCM) formula, which is the refinement of Buyang Huanwu decoction. It has been used in the First Affiliated Hospital of Hunan University of Chinese Medicine for the induction of cerebral ischemia/reperfusion (CIR) injury to promote the recovery of neurological function in patients with CI [7]. Previous studies have shown that HCC extract can protect the neuron population in the hippocampal CA2 region by regulating the expression of iron transporter (Fpn) to balance iron levels after CI [8]. HCC extract also effectively inhibited recombinant human tumor necrosis factor-*α*- (rhT-NF-*α*-) stimulated human umbilical vein endothelial cells (HUVEC) clotting activity, enhanced vWF release, regulated fibrinolytic function, and inhibited PAI activity [9]. In summary, these results indicate that HCC has the potential to treat CI, but its mechanism is still unclear.

Proteomics has developed rapidly in recent years, and its characteristics of studying proteins on a large scale have given opportunities to detect disease targets [10]. The main features of TCM are multidrug, multicomponent, and multitarget features. Meanwhile, CI is also a complex disease, the mechanisms of which are involved in calcium overload, glutamate excitotoxicity, free radical damage, inflammation injury, and energy depletion [11, 12]. These molecular mechanisms are difficult to uncover through a single vertical study; however, proteomics strategies can compensate for the shortcomings of traditional methodologies. Proteomics, as postgenomics, provides a new modern research technology for medical pharmacy research, bringing medicine from microscopic observation of physiological and pathological changes in body tissues to a new stage in which physiological and pathological changes at the molecular level of the proteome of body tissues can be observed [13, 14]. The use of modern science and technology, especially proteomics technology, to study TCM has attracted close attention from the scientific and technological circles at home and abroad. Differential protein and biological mass spectrometry identification techniques used in proteomics have been used in drug efficacy evaluation, and the method principles and techniques are also applicable to TCM evaluation and research [14]. At present, the combination of Liquid Chromatography-tandem mass spectrometry (LC-MS/MS) analysis based on tandem mass tag (TMT) provides an ideal method for proteomics research [13]. Bioinformatics and systematic biological methods also promoted the study of the mechanism of TCM in the treatment of CI [14–16]. Hence, in this study, the quantitative proteomic analysis based on TMT would be used to comprehensively discover the mechanism of HCC intervention in CI.

## 2. Materials and Methods

### 2.1. Drug Preparation

HCC is composed of *Hedysarum multijugum Maxim.* (*Astragali Radix*), *Chuanxiong Rhizoma*, *Pheretima*, *Bombyx Batryticatus* (Specimen number: 2014062101; 2014062410; 2014062205; 20140622307) with ratio 4 : 1:1.5 : 1.5, which was purchased from the Chinese Pharmacy of the First Affiliated Hospital of Hunan University of Chinese Medicine. The herbs were verified by Professor Bing Dai. The herbs were soaked in distilled water, boiled, and fried twice. The two solutions were mixed and concentrated to 1 g of crude drug/ml.

### 2.2. Reagents and Instruments

Reagents were as follows: TMT Labeling Kit (Thermo Inc.), ProteoMiner Low Abundance Protein Enrichment Kit (Bio-Rad Inc.), BCA Kit (Biyuntian Biotechnology Co., Ltd.), trypsin (Promega Inc.), Trifluoroacetic Acid (Sigma-Aldrich Inc.), formic acid (Fluka Inc.), iodoacetamide (Sigma Inc.), dithiothreitol (Sigma Inc.), urea (Sigma Inc.), triethylammonium hydrogen carbonate (Sigma Inc.), ultrapure water (Fisher Chemical Inc.), and 0.26 mm–0.28 mm monofilament nylon fishing line (2838–20A4 Beijing Xion Technology Co., Ltd.). Standard: Ligustrazine Hydrochloride (110817–201608), ferulic acid (110773–201614), and astragaloside IV (110781–201717) were purchased from China Food and Drug Control Research Institute. Rat angiotensinogen ELISA kit (ER0371) and catalase kit (ER0264) were purchased from Wuhan Fine Biotechnology Co., Ltd. Anti-catalase antibody (catalog number ab209211), anti-angiotensinogen antibody (catalog number ab213705), and Anti-beta Actin (catalog number ab8226) were purchased from Abcam company.

Instruments used were as follows: Ultrasonic Cell Breaker (Xinzhi Biotechnology Co., Ltd.), high-speed refrigerated centrifuge, Dynamica microplate reader (Bio-Rad Inc.), and Scientific Q Exactive, Scientific Q Exactive Plus, Scientific Orbitrap Fusion (Thermo Inc.). Column was Welch Vltimate XB-C18 (HS), 4.6 × 250 nm, 5 *μ*m.

### 2.3. Animal Grouping, Modeling, and Intervention Methods

Forty-five (45) specific-pathogen-free (SPF) grade Sprague Dawley (SD) male rats (250–300 g) were purchased from Hunan Slack Jingda Experimental Animal Technology Co., Ltd. [animal license number: SCXK (Xiang) 2013–0004, SCXK (Xiang) 2016–0002]. All rats were housed in cages under controlled environmental conditions (room temperature 23–27°C, relative humidity 40–60%, and 12-hour light/dark cycle). All animals' care and experimental procedures were approved by the Animal Ethics Committee of Hunan University of Chinese Medicine and were in accordance with the National Institute of Health's Guide for the Care and Use of Laboratory Animals.

45 SD rats were randomly divided into three groups: sham operation group (*n* = 15), model group (CI group) (*n* = 15), and HCC group (*n* = 15). The modeling was started 7 days after the adaptive feeding. The modified Zea Longa intracavitary suture blocking method was utilized to ligate the right common carotid artery and external carotid artery of CI model group and HCC group. The fish line with a diameter of 0.20 mm and a smooth spherical shape (0.25–0.30 mm in diameter) was used as an embolism. The right common carotid artery and external carotid artery of the HCC group and the CI group were ligated, and the thread was inserted from the right internal carotid artery to the proximal anterior cerebral artery to block the blood supply of the middle cerebral artery (MCAO). In the sham operation group, only anesthesia and vascular separation were performed, and the blood vessels were not ligated and blocked. After 2 hours of model preparation, the rats in the CI group and the HCC group were scored with the Longa method for 5-point neurological function: 0 points: no obvious neurological symptoms; 1 point: cannot fully extend the left forelimb; 2 points: rotate to the left; 3 points: dump to the left when walking; 4 points: cannot walk by themselves. Those with a score of 1–3 are considered to be successful in model preparation, and the remainder are excluded. The failure model was randomly replaced. After successful modeling, the HCC group were administered with the HCC extracts at 0.5 ml/100 g body weight once daily, while the sham operation group and CI group were administered with the same volume of normal saline once daily. The administration lasted for 7 days.

### 2.4. HPLC Analysis of HCC Extract

The chemical characteristics of HCC extract were determined by HPLC. The mobile phase was a gradient elution system: A: acetonitrile; B: 0.2% phosphoric acid-water; gradient elution flow rate: 1 mL/min; flow rate: 1 ml/min. The process is as follows: 0–15 minutes, 95%–85%; 15–35 minutes, 85–70% A; 35–40 minutes, 70–60% A; 40–50 minutes, 60–60% A; 50–65 minutes, 60–80% A; 70 minutes, 80–95% A; 70–80 minutes, 95–95% A. A photodiode array (PDA) detector was set to detect wavelengths: 198 nm, 201 nm, 280 nm, 290 nm, 315 nm, and 320 nm. Injection volume was 10 *μ*L. The HPLC chromatogram of HCC is shown in Figure S1. The main compounds of HCC were quantified: ligustrazine was 0.36 mg/80 g, ferulic acid was 2.52 mg/80 g, and astragaloside IV was 13.72 mg/80 g.

### 2.5. Pathological Observation

The brain tissue was embedded in paraffin, dehydrated, transparent, and immersed in wax to make a coronal slice of the brain. Then hematoxylin-eosin (HE) staining was performed. After that, the pathomorphological changes were observed under a high-magnification microscope.

BrdU was dissolved in normal saline and injected 100 mg/kg/d into the intraperitoneal cavity of rats. The brain tissue is then subjected to immunofluorescence staining. 10 *μ*L of BrdU monoclonal antibody (1 : 100) was added to a 37°C water bath and incubated. Then the brain tissue was stained with rhodamine (luminescence wavelength 570–590 nm, red light) and bathed in water at 37°C for 30 min. Finally, the slices were packaged with glycerol and observed at 520 and 580 nm through the corresponding color filters with an OLYMPUS BX51 fluorescence microscope.

### 2.6. Proteomics Analysis Method

Under the anesthesia of 1% pentobarbital sodium, the rats were perfused through the heart with ice-cold normal saline and then sacrificed by cervical dislocation; and then the serum was obtained. After the ProteoMiner low-abundance protein enrichment kit removes the high-abundance protein in the serum sample, the BCA kit is used to determine the protein concentration of the eluted low-abundance protein. Then trypsin digestion was carried out, and the peptides were labeled according to the operating instructions of the TMT kit. Then, the labeled peptides were separated by the EASY-nL C 1000 ultra-high-performance liquid system and then analyzed using the Orbit rap Fusion Lumos mass spectrometer. After that, Maxquant (v1.5.2.8) was used to search the MS mass spectrum data. Fold change >1.3 was considered to be significantly upregulated, while fold change <0.77 was considered to be significantly downregulated. False discovery rate (FDR) < 0.05 was considered as differential expression.

### 2.7. Bioinformatics Analysis Methods

The UniProt IDs of the DEPs were input into David database (https://david-d.ncifcrf.gov, ver. 6.8) [17] and the Metascape database (http://metascape.org/gp/index.html#/main/step1) [18] to undergo GO and signaling pathway enrichment analysis. Protein-protein interaction (PPI) was collected from STRING 10.0 (http://www.string-db.org/) [19].

### 2.8. Detection of Serum Angiotensinogen and Catalase Protein by ELISA

The rat serum was centrifuged (3000 r/min) for 15 minutes, and the upper serum was collected, and then the angiotensinogen and catalase levels were detected according to the ELISA kit instructions. The microplate reader was used to detect the optical density (OD) value of the sample at a wavelength of 450 nm.

### 2.9. Detection of Angiotensinogen and Catalase Protein by Western Blot

Angiotensinogen and catalase protein were detected by Western blotting to verify the proteomics data. The original serum without high-abundance protein was quantified by Brad Ford method, and the appropriate amount of sample was selected by calculation and mixed thoroughly with the loading buffer, and the protein was denatured at 95°C for 10 min. After gel electrophoresis, the protein was transferred to the PVDF membrane after SDS-polyacrylamide gel electrophoresis. After 5% skim milk was blocked for 2 hours, anti-catalase antibody and anti-angiotensinogen antibody were added and incubated at 4°C for 2 hours. Finally, the luminescent solution was added for development, and the Bio-Rad automatic gel imaging system took pictures with *β*-actin as an internal reference. QuantityOne v4.6.7 software was used to process the results of Western blotting experiment, and the corresponding gray value of the protein band was used as the internal reference for data correction.

### 2.10. Statistical Analysis

The measurement data were expressed as mean ± standard deviation (SD). The neurological function score (mNSS score) of the rats was analyzed by one-way analysis of variance using SPSS software 19.0. *P* < 0.05 was considered to be statistically significant.

## 3. Results

### 3.1. Neuroprotective Effects of HCC and Pathological Changes of Brain Tissue

The neurological function scores of the HCC group were lower than those of the CI group, indicating that HCC can improve neurological function after CI (Table 1).

**Figure 1:**
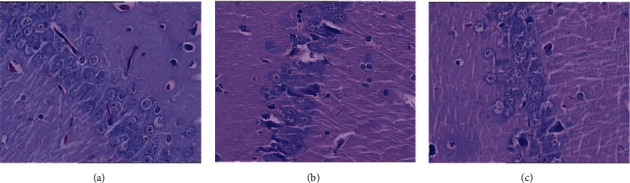
Pathological changes of brain tissue (HE staining, 400X. (a) sham operation group; (b) model group; (c) HCC group).

HE staining showed that the neurons in the hippocampal CA1 area of the sham operation group were clearly and neatly arranged, with large and round nuclei and uniform cytoplasmic staining. The number of cells in the model group decreased, the cells were not aligned, the gap increased, and the nucleus was densely condensed. The HCC group had more cell layers than the model group, and a small amount of nucleus shrinkage and cytoplasm staining were still seen (Figure 1).

**Figure 2:**
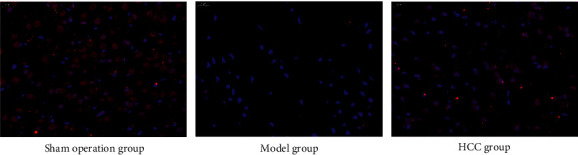
Pathological changes of brain tissue (immunofluorescence staining, 400X).

Immunofluorescence showed that BrdU signal appeared in HCC group, which can be considered as new nerve cells. The number of positive signals in the HCC group was higher than that in the model group, indicating that the number of new nerve cells increased after HCC treatment (Figure 2).

### 3.2. Differential Protein Analysis

**Figure 3:**
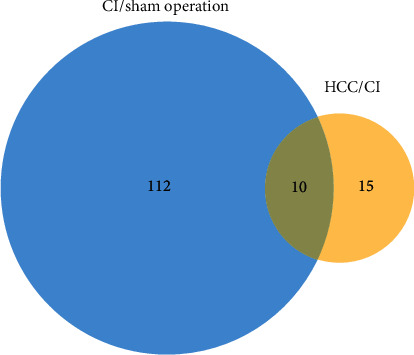
Venn diagram of DEPs.

A total of 1340 proteins were identified in this study, of which 1138 proteins contain quantitative information with an FDR of less than 1%. There are 122 DEPs (92 upregulated proteins and 30 downregulated proteins) in the CI/sham operation group and 25 DEPs (12 upregulated proteins and 13 downregulated proteins) in the HCC/CI group. There are 12 upregulated proteins in HCC/CI group (Table S1). The intersection of DEPs in the CI/sham operation group and DEPs in the HCC/CI group includes 10 proteins (Figure 3). The 12 upregulated proteins in HCC/CI group include Protein Actn2, Kelch-like protein 41, Alpha-1,4 glucan phosphorylase, Protein Lrtm2, Dystrophin, Galectin-1, C4b-binding protein beta chain, and so on. The 13 downregulated proteins include Alpha-2 antiplasmin, Arachidonate 15-lipoxygenase, Carbonic anhydrase 2, Complement factor I, angiotensinogen, catalase, Protein LOC103691744, Anionic trypsin-1, and so on.

### 3.3. DEPs PPI Network Analysis

**Figure 4:**
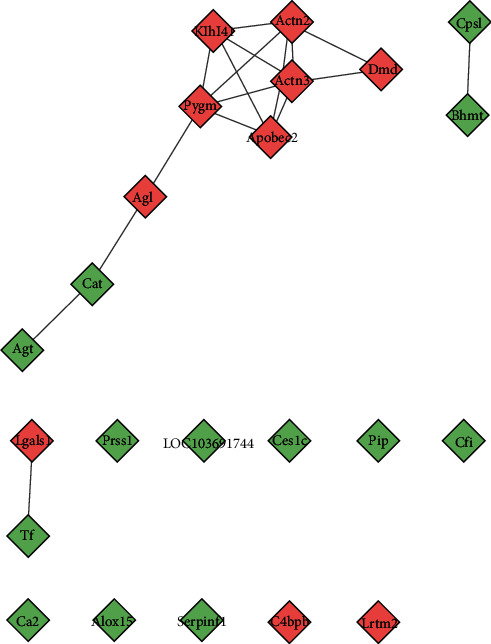
DEPs PPI network (green diamond stands for downregulated DEPs; red diamond stands for upregulated DEPs).

**Figure 5:**
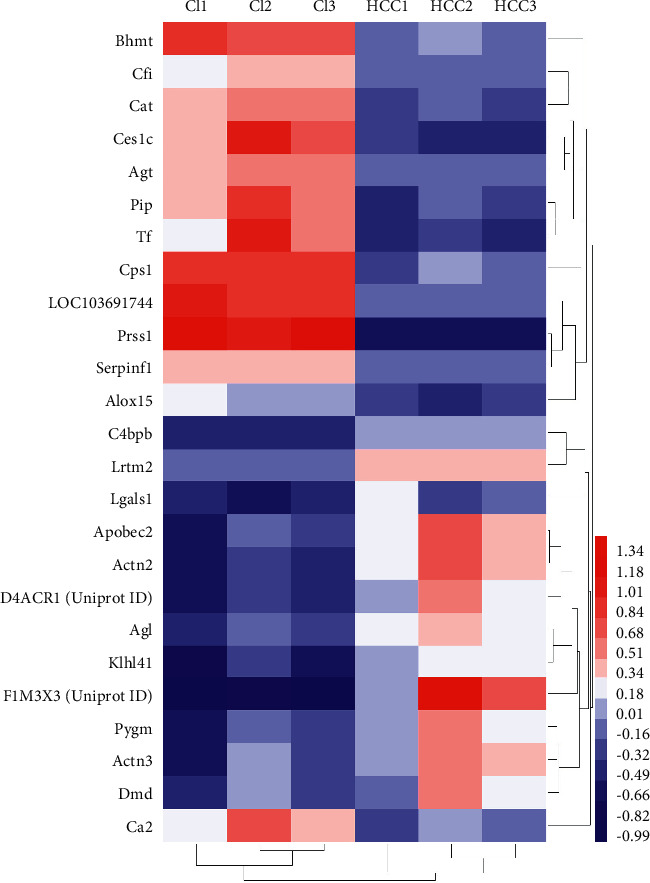
Cluster heatmap of DEPs in the HCC/CI group [color indicates Log2 (fold change) of proteins].

**Figure 6:**
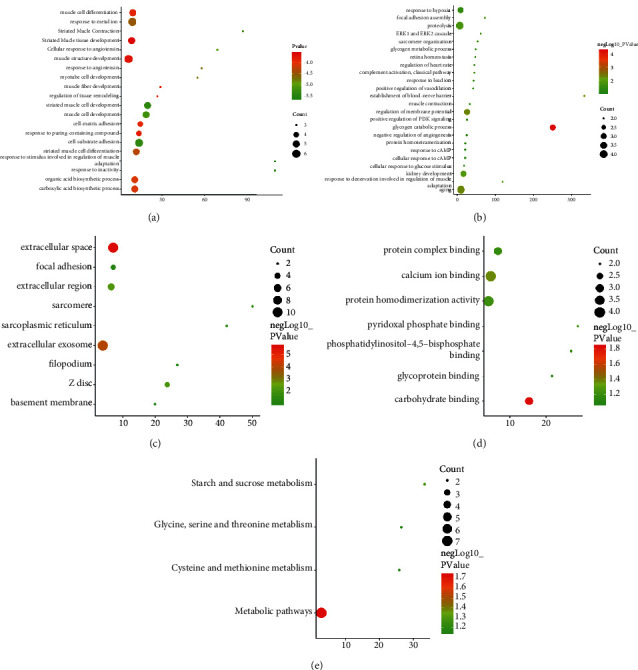
GO enrichment analysis of DEPs ((a) top 20 biological processes from Metascape; (b) biological processes from David; (c) cellular components; (d) molecular functions; (e) signaling pathways. *X*-axis stands for fold enrichment).

**Figure 7:**
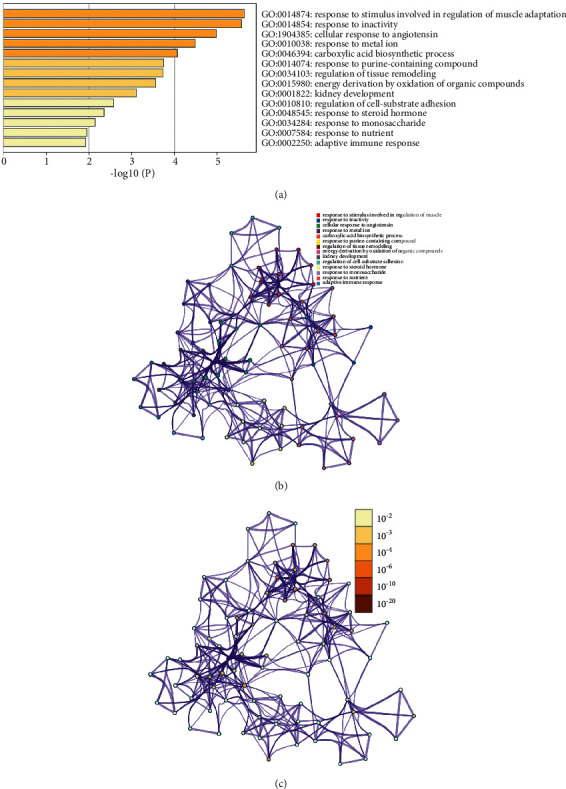
Enrichment analysis results in Metascape ((a) Heatmap of top 14 biological processes; (b) PPI network colored by cluster; (c) PPI network colored by *P*-value).

The DEPs PPI network was shown in Figure 4. This network showed that 13 DEPs could directly interact with each other, while the other 12 DEPs did not show any direct interaction. The degrees of upregulated DEPs that can interact were Actn2 (5 edges), Actn3 (5 edges), Pygm (5 edges), Klhl41 (4 edges), Apobec2 (4 edges), Dmd (2 edges), Agl (2 edges), and Lgals1 (1 edge); those of downregulated DEPs were Cat (2 edges), Bhmt (1 edge), Cps1 (1 edge), Agt (1 edge), and Tf (1 edge). The cluster heatmap of 25 DEPs in the HCC/CI group was shown in Figure 5. In order to understand the relationship between the affected DEPs during the treatment of HCC, 25 DEPs from the HCC/CI group were imported into David database and metascape database for enrichment analysis. The *P*-value, fold enrichment, gene count of each biological process, cellular components, molecular functions, and signaling pathways were shown in Figure 6. The heatmap of the top 14 biological processes, PPI network colored by cluster, and PPI network colored by *P* value were shown in Figure 7. The result of Metascape showed that the functions of DEPs are mainly focused on cell-substrate adhesion and regulation, reactive oxygen species metabolic process, angiotensin response (cellular response to angiotensin, response to angiotensin), positive regulation of the occurrence of nerves and neurons (positive regulation of neurogenesis), inflammatory response, response to hypoxia (response to hypoxia, response to decreased oxygen levels), and cellular calcium homeostasis (cellular calcium ion homeostasis).

**Figure 8:**
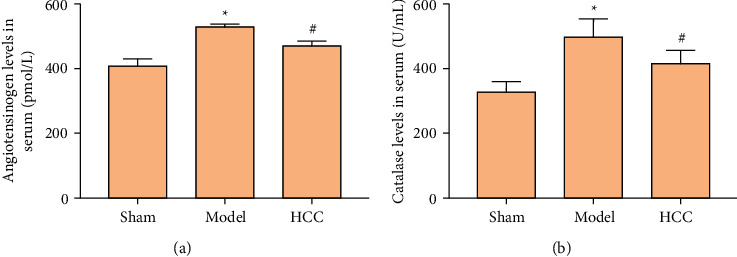
Serum angiotensinogen and catalase level ((a) angiotensinogen; (b) catalase. ^*∗*^Compared with sham operation group, *P* < 0.05; #compared with model group, *P* < 0.05).

The result of David showed that the biological processes regulated by HCC are glycogenolysis process, aging, establishing a blood-nerve barrier, reaction, glycogen metabolism, complement activation, and classical pathway. The cellular components regulated by HCC are extracellular space, extracellular bodies, extracellular regions, and sarcoplasmic reticulum. The molecular functions regulated by HCC are carbohydrate binding and calcium ion binding. The signaling pathway regulated by HCC is the metabolic pathway (Figure 8).

### 3.4. DEPs Expression in Brain Tissue

The expression of DEPs in each organ in each comparison group was analyzed, and the obtained DEPs were analyzed by Expression Atlas. After the tissue specificity was set to “brain,” the protein number greater than or equal to 10 was matched in turn. These proteins are sorted according to the specificity of brain tissue (see Tables 2 and 3 for details).

DEPs with upregulated expression and high brain tissue specificity in CI/sham operation comparison group are Fructose-bisphosphate aldolase A, Elongation factor 2, Serotransferrin, “ATPase, H+ transporting, V1 subunit E isoform 1, isoform CRA_a”, 14-3-3 protein epsilon, 60S acidic ribosomal protein P0, phosphoglycerate kinase 1, Pyruvate kinase, Endoplasmin, and ATPase H+ -transporting V1 subunit A. And DEPs with downregulated expression and high brain tissue specificity in CI/sham operation comparison group are Tubulin alpha-4A chain, Ras-related protein Rap-1b, Insulin-like growth factor-binding protein 2, Galectin-1, cAMP-dependent protein kinase type II-beta regulatory subunit, Tropomyosin alpha-4 chain, 40S ribosomal protein S6, Acylamino-acid-releasing enzyme, and Ras suppressor protein 1.

DEPs with upregulated expression and high brain tissue specificity in HCC/CI comparison group were Galectin-1, Dystrophin, Alpha-1, 4 glucan phosphorylase, Protein Lrtm2, and Actinin alpha 2. DEPs with downregulated expression and high brain tissue specificity in HCC/CI comparison group were Serotransferrin, Carbonic anhydrase 2, angiotensinogen, and catalase.

### 3.5. Serum Angiotensinogen and Catalase Level Detected by ELISA

The results of ELISA showed that, compared with sham operation group, the angiotensinogen and catalase in model group were increased (*P* < 0.05). Compared with model group, the angiotensinogen and catalase in HCC group were decreased (*P* < 0.05). This is consistent with the findings of proteomics (Figure 8).

### 3.6. Serum Angiotensinogen and Catalase Level Detected by Western Blotting

**Figure 9:**
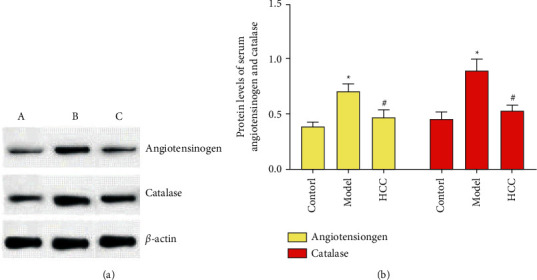
Serum angiotensinogen and catalase level ((a) sham operation group; (b) model group; (c) HCC group. ^*∗*^Compared with sham operation group, *P* < 0.05; #compared with model group, *P* < 0.05).

The results of Western blotting showed that, compared with sham operation group, the angiotensinogen and catalase in model group were increased (*P* < 0.05). Compared with model group, the angiotensinogen and catalase in HCC group were decreased (*P* < 0.05). This is consistent with the findings of proteomics (Figure 9).

## 4. Discussion

HCC is composed of *Astragali Radix, Chuanxiong Rhizoma, Pheretima, Bombyx Batryticatus*. The main medicinal ingredients of *Chuanxiong Rhizoma* are ligustrazine, ligustrazine, folic acid, and so on. The main medicinal ingredients of *Pheretima* are cholesterol ferulic acid, guanosine, coccinea glycoside, and so on. The main medicinal ingredients of *Bombyx Batryticatus* are Beauverin, Beauveria yellow pigment, and so on. *Astragali Radix* can be divided into flavonoids, polysaccharides, saponins, alkaloids, and pyrazines. These ingredients have certain pharmacological effects. For example, the flavonoids in *Astragali Radix* can regulate immunity, inhibit inflammation and oxidative stress, and have antitumor activity [20–23]. Saponins can improve CI by regulating biological processes such as energy metabolism disorder, depolarization around the infarct, oxidative stress damage, inflammatory response, and apoptosis in CI [24–26]. Alkaloid components and pyrazine components play a role in scavenging free radicals and antioxidation by downregulating immune cells and inflammatory factors, so as to reduce brain damage [27–29]. This study found that HCC may treat CI by regulating some CI-related proteins, such as Prs1, Cps1, LOC103691744, Bhmt, Pip, angiotensinogen, Cat, Serpinf1, Tf, and Lgals1.

CI mainly results from thrombotic vascular occlusion. The increased *α*2-antiplasmin (*α*2-AP) level is associated with an increased risk of CI, which could lead to failure of TPA therapy [30–32]. Researchers found that *α*2-AP neutralized the therapeutic benefit of TPA therapy [33]. In addition, in the absence of treatment, the ischemic injury of *α*2-AP to the brain is dose-dependent [33]. Inhibition of *α*2-AP production can significantly reduce microvascular thrombosis, ischemic brain injury, cerebral thromboembolic swelling after stroke, cerebral hemorrhage, and death [30]. In short, *α*2-AP is very important in the pathogenesis of ischemic brain injury. Our study showed that *α*2-AP was significantly increased in CI group, and it was downregulated after the intervention of HCC, suggesting that HCC may downregulate *α*2-AP and inhibit blood coagulation, thereby reducing ischemic damage to the brain. In our previous research, HCC extract can effectively inhibit rhVEGF-*α*-stimulated HUVEC clotting activity, enhance vWF release, regulate fibrinolytic function, and inhibit PAI activity [9].

Serum transferrin is an iron-binding transporter that transports iron from the site of absorption and heme degradation to the site of storage and utilization. Meanwhile, serum transferrin may also stimulate cell proliferation. Carotid atherosclerotic plaques have significantly high levels of infiltration of inflammatory macrophages and high-iron-related lesions [8]. Serum transferrin is significantly elevated in the occurrence of CI, which may lead to deposition of iron in cells. After HCC intervention, transferrin was downregulated, indicating that HCC may reduce iron deposition in cells by downregulating transferrin, thereby reducing ischemic damage in the brain [8].

Angiotensinogen is an essential component of the renin-angiotensin system (RAS), which is a potent regulator of blood pressure, body fluids, and electrolyte homeostasis. Angiotensin 1–7 is a ligand of G protein-coupled receptor MAS1, which has vasodilatation, antidiuretic, and antithrombotic effects [34]. In addition, in human renin and angiotensinogen double transgenic mice, the activation of human Ras enhances ischemia-induced brain injury by significantly reducing cerebral blood flow and enhancing oxidative stress [35, 36]. Our study shows that HCC may promote blood supply in infarcted areas by lowering angiotensinogen in serum and thereby relaxing blood vessels after CI.

Catalase protects cells from the toxic effects of hydrogen peroxide in almost all aerobic organisms, which can also promote cell growth [37]. The increase in catalase indicates the presence of oxidation in tissues or cells. The expression of catalase in CI rats was downregulated after HCC intervention, suggesting that HCC may reduce oxidative stress damage induced by CI.

Current research showed that galectin (*β*-galactoside-binding lectin) plays multiple roles in the regulation of immune and inflammatory responses. In neuronal diseases and different experimental neuroinflammatory disease models, galectin can act as an extracellular medium or intracellular regulator to control the inflammatory response or confer remodeling ability to the damaged neural tissue. In CI model, Galectin-1 regulates neurogenesis in the subventricular zone and promotes functional recovery after stroke [38, 39]. In vitro studies found that lectin-1 can promote the expression and secretion of brain-derived neurotrophic factor in astrocytes cultured in hypoxic serum [40]. In this research, Galectin-1 was downregulated in the CI group, while it was upregulated after HCC intervention. This suggests that HCC may promote neuroprotection after CI by regulating the protein, thereby reducing the ischemia-anoxia injury caused by CI.

The DEPs found in the experiment showed different trends in the CI group and the HCC group, indicating that the drug has an effect on its expression, which can speculate that these DEPs may be the target of HCC treatment of CI. In addition, although the pathological changes of the CI are mainly in the brain, the targets of drug are not limited to the local part of the brain tissue. We found that HCC may achieve therapeutic effects by regulating other targets outside the brain. Therefore, the mechanism by which HCC treats CI may be the result of multiple target interactions. This study provides proteomic evidence for the protective role of HCC in CI. However, the study has limitations. The advantages of this research are as follows: (1) the previous proteomics studies of TCM intervention in CI mainly focused on the areas of brain tissue and cerebrospinal fluid [41, 42], while this article focused on the proteomics profile of serum, supplementing this key link. (2) The previous techniques used in proteomics were mainly two-dimensional gel electrophoresis. This study uses the latest TMT-labeled proteomics, which has greater detection accuracy and is conducive to the discovery of new ischemic stroke markers and therapeutic markers after HCC intervention. The limitations of this research are as follows: (1) the protective mechanism of HCC against cerebral infarction is explored only through serum proteomics. Therefore, we will further conduct proteomic research on the tissues of cerebral infarction in the future. In addition, we have found that oxidative stress is the main mechanism of HCC in the treatment of CI. In the future, we will in-depth study the molecular mechanism of HCC and its medicinal components in the treatment of CI in terms of oxidative stress. Hence, the mechanism of oxidative stress and ferroptosis involved is still the focus of our next research. (2) The TMT proteomics technology we use is currently not the latest proteomics technology. In the future, we would further improve the pharmacodynamic basis of HCC intervention in CI through systems pharmacology [11], computer network pharmacology [12], pharmacokinetics, and more refined proteomics (such as TMT proteomics technology).

## 5. Conclusion

HCC may treat CI through regulating cell-substrate adhesion and regulation, reactive oxygen species metabolic process, angiotensin response (cellular response to angiotensin, response to angiotensin), positive regulation of the occurrence of nerves and neurons (positive regulation of neurogenesis), inflammatory response, response to hypoxia (response to hypoxia, response to decreased oxygen levels), cellular calcium homeostasis (cellular calcium ion homeostasis), and so on. This study provides new reference for the clinical application of HCC for CI.

## Figures and Tables

**Table 1:** Neurological function scores (*X* ± *S*, *n* = 15).

Groups	Score
Sham operation	0
CI	2.813 ± 0.403
HCC	2.185 ± 0.870^*∗*^

^*∗*^compared with the model group. *P* < 0.05.

**Table 2:** Top 10 DEPs with higher expression in brain tissue in the CI/sham operation comparison group.

Gene name	Adrenal gland	Brain	Gastrocnemius	Heart	Kidney	Liver	Lung	Spleen	Testis	Thymus
Aldoa	566	717	7423	730	377	37	590	173	331	362
Eef2	565	488	531	318	377	499	855	1446	615	1004
Tf	42	447	41	24	43	18878	109	126	127	41
Atp6v1e1	168	288	66	52	126	29	83	61	44	76
Ywhae	133	282	122	63	99	85	160	137	161	189
Rplp0	536	276	507	340	490	421	1131	2620	566	1629
Pgk1	274	270	538	156	218	158	134	145	22	158
Pkm	118	247	641	110	32	5	89	57	69	94
Hsp90b1	120	182	50	66	169	278	249	290	287	153
Atp6v1a	42	176	10	8	93	22	52	37	15	33

**Table 3:** Top 10 DEPs with higher expression in brain tissue in the HCC/CI comparison group.

Gene name	Adrenal gland	Brain	Gastrocnemius	Heart	Kidney	Liver	Lung	Spleen	Testis	Thymus
Lgals1	311	37	254	156	42	6	274	79	65	161
Dmd	8	31	66	58	3	6	20	11	7	4
Pygm	3	25	2485	208	3	2	68	7	2	9
Lrtm2	3	16	1	1	3	42	4	3	2	5
Actn2	14	10	651	440	0.8	6	55	3	1	7
Tf	42	447	41	24	43	18878	109	126	127	41
Car2	27	183	12	11	250	6	101	293	40	12
Angiotensinogen	47	96	2	2	13	372	31	6	0.7	51
Cat	146	21	48	46	379	1270	113	81	8	56

## Data Availability

The data used to support the findings of this study are included within the article.

## Supplementary Materials

Supplementary Materials Supplementary Materials. Figure S1: the HPLC results. Table S1: protein accession, name, and multiple of difference of DEPs.
