# Supplementary material for: Uncovering the Neuroprotective Effect of Hedysarum multijugum Maxim-Chuanxiong Rhizoma Compound on Cerebral Infarction through Quantitative Proteomics
Source: Evid Based Complement Alternat Med. 2022 Mar 26;2022:5241902. doi: 10.1155/2022/5241902 (PMC8976648; doi:10.1155/2022/5241902)
Supplement: Supplementary Materials — Figure S1: the HPLC results. Table S1: protein accession, name, and multiple of difference of DEPs. [file 5241902.f1.zip › 5241902.f1/Table S1 (1).pdf]

Table S1 Protein accession, name and multi

Group	Protein accession
	P00762
	P07756
	A0A0G2K5G2
	A0A0G2JSK9
	Q923Z2
	P58775
	P68136
	G3V8B0
	G3V6E1
	A0A0G2JSP8
	P04692
	P04466
	D4A4D5
	P06757
	D3ZZT9
	Q6P725
	Q6AYW2
	F1M853
	F1MAA7
	G3V7U4
	P63018
	A0A0G2K484
	G3V812
	G3V8L3
	P19945
	G3V7K1
	D3ZCI0
	P02600
	Q68FY4
	A0A0H2UHR7
	F1LRV9
	P28037
	P05065
	A0A0H2UHM3
	A0A0H2UHJ1
	P06214
	C0JPT7
	B0BMS8
	F7ESM5
	F1LZ34
	Q68FW9
	D3ZXY4
	O35567
	P01015
	P63039
	A0A0G2K5P5
	D3ZY96
	P10760
	A0A0G2K0S8
	F1LRT1
	P09811
	G3V9G4
	A0A0G2JSI1

CI / sham operation group	P04762
	A0A0G2JVG3
	Q6P6S4
	A0A140TAI1
	P05197
	A0A0G2JW80
	Q8CG45
	Q99MH3
	A0A0G2K542
	A0A0H2UHI5
	Q63083
	P62260
	Q4QRB8
	P82995
	A0A0G2JWK7
	Q9Z0V5
	P16617
	P68035
	O35509
	Q9JKB7
	G3V9S9
	A0A0G2JZS2
	P57756
	P50115
	F1LRV4
	Q80ZA3
	Q4V8H5
	P12346
	A0A0G2JYL4
	Q64604
	Q68FS2
	F1M614
	Q6AYS3
	P04785
	A0A0A0MY09
	G3V8L7
	A0A0G2JSY2
	G3V7L8
	D4A133
	O55004
	D4A526
	D3ZD40
	F1LWS4
	C0M4B0
	D3ZRX9
	A0A0G2K975
	A0A0G2JVP4
	A0A0G2K3W2
	Q62636
	Q5XIF6
	M0R5R0
	D4A8F2
	P12843
	G3V9E4
	A0A0G2K5E2
	P09495

	Q4KM33
	A0A0G2JX40
	G3V8X6
	P11762
	P14423
	P21743
	F1M1R0
	M0RD75
	F1MAE7
	Q62740
	P12369
	Q4QQS0
	D3ZZ08
	F1M3X3
	D3ZCV0
	B4F789
	Q9ER30
	D4ACR1
	Q8R4I6
	D4AEH9
	G3V8V3
	D3ZPP7
	P11530
	P11762
	A0A5C5
HCC/CI group	Q80ZA3
	Q02759
	P27139
	A0A0G2K135
	P01015
	P04762
	A0A0G2JSK9
	G3V812
	P12346
	P07756
	D3ZGK7
	A0A0G2K5G2
	P00762

ple of difference of DEPs

Protein description	Gene name	Ratio
Anionic trypsin-1	Prss1	4.631
"Carbamoyl-phosphate synthase [ammonia], mitochondrial"	Cps1	4.184
Protein LOC103691744	LOC103691744	4.174
Betaine--homocysteine S-methyltransferase 1	Bhmt	4.005
"Tropomyosin 1, alpha, isoform CRA_a"	Tpm1	3.376
Tropomyosin beta chain	Tpm2	3.257
"Actin, alpha skeletal muscle"	Acta1	3.221
Myosin-7	Myh7	2.873
Protein Myh1	Myh2	2.763
Creatine kinase M-type	Ckm	2.532
Tropomyosin alpha-1 chain	Tpm1	2.41
"Myosin regulatory light chain 2, skeletal muscle isoform"	Mylpf	2.39
Protein LOC100362751	LOC498555	2.379
Alcohol dehydrogenase 1	Adh1	2.348
Protein Col14a1	Col14a1	2.295
Desmin	Des	2.285
Phenylalanine hydroxylase	Pah	2.261
Protein Rrbp1	Rrbp1	2.254
Protein Lamc1	Lamc1	2.232
Lamin-B1	Lmnb1	2.227
Heat shock cognate 71 kDa protein	Hspa8	2.161
Protein Myh1	Myh1	2.155
"Prolactin induced protein, isoform CRA_d"	Pip	2.152
"Lamin A, isoform CRA_b"	Lmna	2.146
60S acidic ribosomal protein P0	Rplp0	2.136
Myomesin 2	Myom2	2.09
Glycerol kinase	Gk	2.08
"Myosin light chain 1/3, skeletal muscle isoform"	Myl1	2.052
Group specific component	Gc	2.016
Filamin-C	Flnc	1.991
Protein Myh1	Myh1	1.987
Cytosolic 10-formyltetrahydrofolate dehydrogenase	Aldh1l1	1.968
Fructose-bisphosphate aldolase A	Aldoa	1.927
Haptoglobin	Hp	1.9
Protein S100-A9	S100a9	1.873
Delta-aminolevulinic acid dehydratase	Alad	1.858
Filamin alpha	Flna	1.81
Myl9 protein	Myl9	1.792
"Nitrilase 1, isoform CRA_a"	/	1.789
S-adenosylmethionine synthase	Mat1a	1.787
COP9 signalosome complex subunit 3	Cops3	1.787
Protein Aldh8a1	Aldh8a1	1.784
Bifunctional purine biosynthesis protein PURH	Atic	1.777
Angiotensinogen	Agt	1.777
"60 kDa heat shock protein, mitochondrial"	Hspd1	1.749
Protein Myom1	Myom1	1.745
Neutrophilic granule protein (Predicted)	Ngp	1.729
Adenosylhomocysteinase	Ahcy	1.729
"6-phosphofructo-2-kinase/fructose-2,6-bisphosphatase 1"	Pfkfb1	1.723
"Fructose-1,6-bisphosphatase 1"	Fbp1	1.709
"Glycogen phosphorylase, liver form"	Pygl	1.704
ATP-citrate synthase	Acly	1.686
4-trimethylaminobutyraldehyde dehydrogenase	Aldh9a1	1.67

Catalase	Cat	1.669
Pyruvate kinase	Pkm	1.667
Nucleotide exchange factor SIL1	Sil1	1.666
"Ubiquilin 1, isoform CRA_a"	Ubqln1	1.658
Elongation factor 2	Eef2	1.652
COP9 signalosome complex subunit 1	Gps1	1.65
Aflatoxin B1 aldehyde reductase member 2	Akr7a2	1.647
Hepcidin	Hamp	1.643
Protein Ugp2	Ugp2	1.635
Protein LOC299282	Serpina3n	1.632
Nucleobindin-1	Nucb1	1.625
14-3-3 protein epsilon	Ywhae	1.607
Argininosuccinate lyase	Asl	1.607
Heat shock protein HSP 90-alpha	Hsp90aa1	1.606
Transgelin	Tagln	1.605
Peroxiredoxin-4	Prdx4	1.601
Phosphoglycerate kinase 1	Pgk1	1.581
"Actin, alpha cardiac muscle 1"	Actc1	1.579
Ras-related protein Rab-11B	Rab11b	1.566
Guanine deaminase	Gda	1.566
Protein Sec24d	Sec24d	1.551
Polyadenylate-binding protein 1	Pabpc1	1.544
Ficolin-2	Fcn2	1.54
Protein S100-A8	S100a8	1.535
Heat shock 70 kDa protein 4	Hspa4	1.526
Alpha-2 antiplasmin	Serpinf1	1.488
Aspartyl aminopeptidase	Dnpep	1.473
Serotransferrin	Tf	1.471
Protein P4ha2	P4ha2	1.457
Receptor-type tyrosine-protein phosphatase F	Ptprf	1.443
COP9 signalosome complex subunit 4	Cops4	1.441
Protein Lama2	Lama2	1.429
Carboxypeptidase	Ctsa	1.415
Protein disulfide-isomerase	P4hb	1.408
Endoplasmin	Hsp90b1	1.395
Integrin alpha M	Itgam	1.393
Calpastatin	Cast	1.368
"ATPase, H+ transporting, V1 subunit E isoform 1, isoform CRA_a"	Atp6v1e1	1.351
Protein Atp6v1a	Atp6v1a	1.341
Ribonuclease 4	Rnase4	0.762
Leukocyte cell-derived chemotaxin 2 (Predicted)	Lect2	0.739
Protein Papln	Papln	0.734
Uncharacterized protein	/	0.734
Matrix metalloproteinase 19	Mmp19	0.724
Calponin	Cnn2	0.714
Protein RGD1564614	RGD1564614	0.711
Protein Ighm	/	0.707
Protein F5	F5	0.692
Ras-related protein Rap-1b	Rap1b	0.691
Tubulin alpha-4A chain	Tuba4a	0.683
"Protein S (Alpha), isoform CRA_b"	Pros1	0.679
Protein Rsu1	Rsu1	0.675
Insulin-like growth factor-binding protein 2	Igfbp2	0.661
Acylamino-acid-releasing enzyme	Apeh	0.65
Uncharacterized protein	Scarf1	0.649
Tropomyosin alpha-4 chain	Tpm4	0.647

Pleckstrin	Plek	0.638
Insulin-like growth factor I	Igf1	0.632
"RCG39455, isoform CRA_a"	Tsku	0.602
Galectin-1	Lgals1	0.599
"Phospholipase A2, membrane associated"	Pla2g2a	0.562
Insulin-like growth factor-binding protein 1	Igfbp1	0.56
Protein Igkv8-27	/	0.536
40S ribosomal protein S6	Rps6	0.535
Uncharacterized protein	/	0.53
Secreted phosphoprotein 24	Spp2	0.521
cAMP-dependent protein kinase type II-beta regulatory subunit	Prkar2b	0.506
Interleukin-1 receptor-associated kinase-like 2	Irak2	0.447
Uncharacterized protein	/	0.395
Uncharacterized protein	/	3.466
Protein Actn2	Actn2	1.763
"Apolipoprotein B editing complex 2 (Predicted), isoform CRA_a"	Apobec2	1.728
Kelch-like protein 41	Klhl41	1.706
Uncharacterized protein	/	1.59
"Actinin alpha 3, isoform CRA_a"	Actn3	1.56
"Amylo-1, 6-glucosidase, 4-alpha-glucanotransferase (Glycogen debranc	Agl	1.511
"Alpha-1,4 glucan phosphorylase"	Pygm	1.508
Protein Lrtm2	Lrtm2	1.464
Dystrophin	Dmd	1.396
Galectin-1	Lgals1	1.391
C4b-binding protein beta chain	C4bpb	1.333
Alpha-2 antiplasmin	Serpinf1	0.748
Arachidonate 15-lipoxygenase	Alox15	0.743
Carbonic anhydrase 2	Ca2	0.715
Complement factor I	Cfi	0.704
Angiotensinogen	Agt	0.684
Catalase	Cat	0.613
Betaine--homocysteine S-methyltransferase 1	Bhmt	0.58
"Prolactin induced protein, isoform CRA_d"	Pip	0.535
Serotransferrin	Tf	0.505
"Carbamoyl-phosphate synthase [ammonia], mitochondrial"	Cps1	0.498
Carboxylic ester hydrolase	Ces1c	0.481
Protein LOC103691744	LOC103691744	0.478
Anionic trypsin-1	Prss1	0.281

Differential expression
Up
Up
Up
Up
Up
Up
Up
Up
Up
Up
Up
Up
Up
Up
Up
Up
Up
Up
Up
Up
Up
Up
Up
Up
Up
Up
Up
Up
Up
Up
Up
Up
Up
Up
Up
Up
Up
Up
Up
Up
Up
Up
Up
Up
Up
Up
Up
Up
Up
Up
Up
Up
Up

Up
Up
Up
Up
Up
Up
Up
Up
Up
Up
Up
Up
Up
Up
Up
Up
Up
Up
Up
Up
Up
Up
Up
Up
Up
Up
Up
Up
Up
Up
Up
Up
Up
Up
Up
Up
Up
Up
Up
Down
Down
Down
Down
Down
Down
Down
Down
Down
Down
Down
Down
Down
Down
Down
Down
Down

Down
Down
Down
Down
Down
Down
Down
Down
Down
Down
Down
Down
Down
Up
Up
Up
Up
Up
Up
Up
Up
Up
Up
Up
Up
Down
Down
Down
Down
Down
Down
Down
Down
Down
Down
Down
Down
Down
