# Supplementary figures and images for: Uncovering the Neuroprotective Effect of Hedysarum multijugum Maxim-Chuanxiong Rhizoma Compound on Cerebral Infarction through Quantitative Proteomics
Source: Evid Based Complement Alternat Med. 2022 Mar 26;2022:5241902. doi: 10.1155/2022/5241902 (PMC8976648; doi:10.1155/2022/5241902)

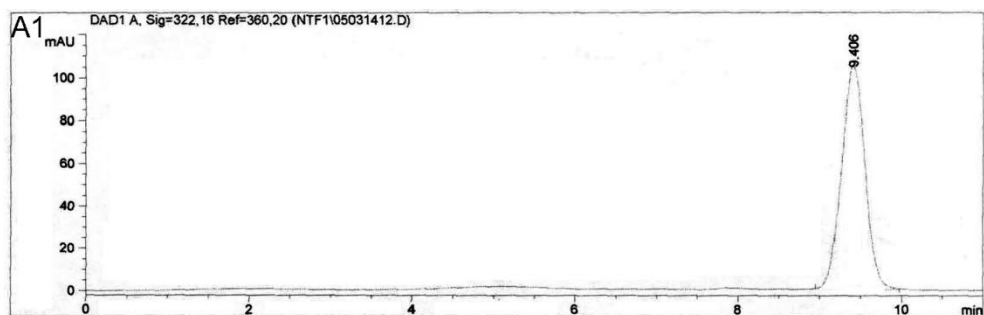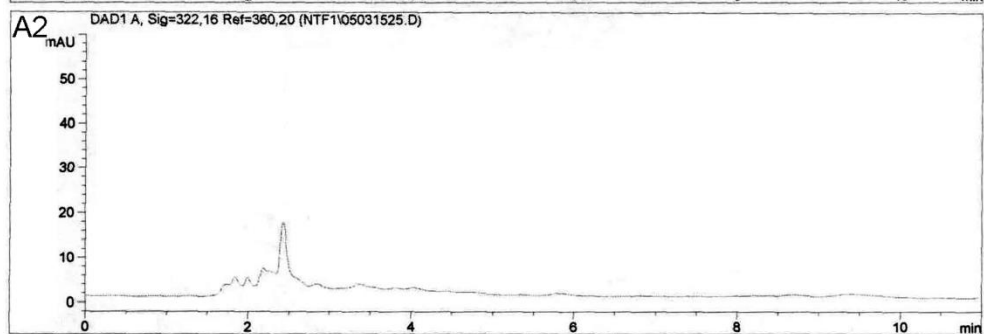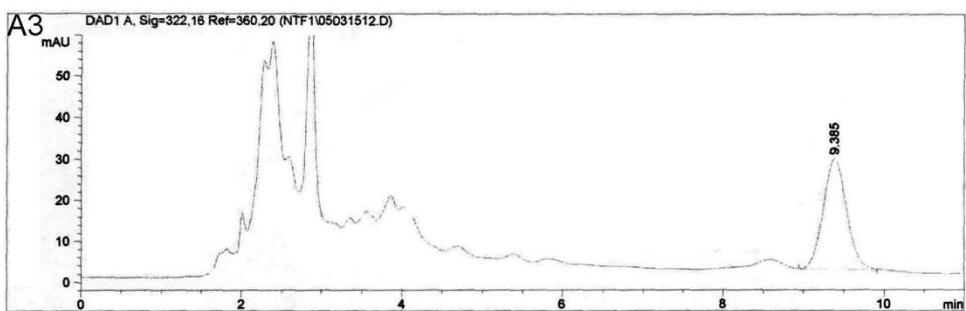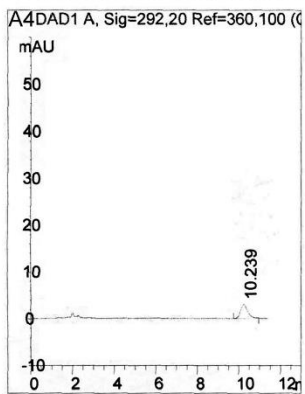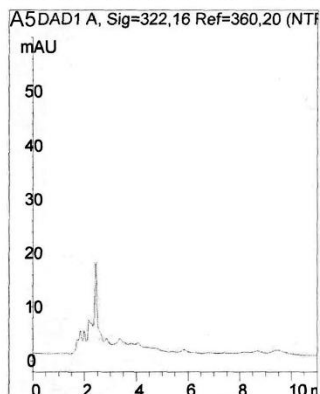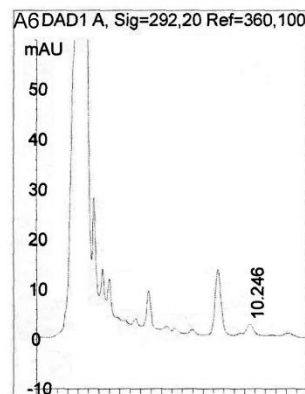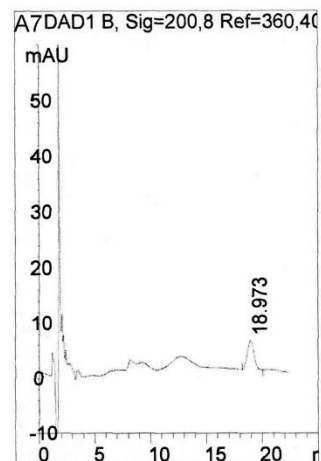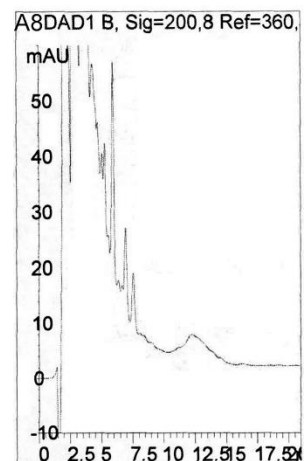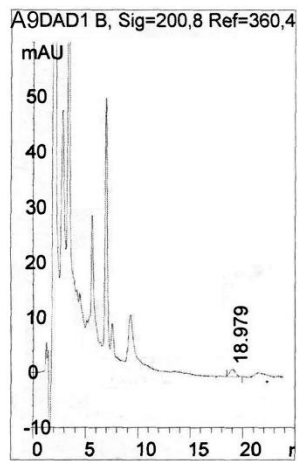

Figure S1 The results of HPLC (A1-A3: Ferulic acid; A4-A6: Ligustrazine; A7-A9: Astragaloside IV)

Supplement: Supplementary Materials — Figure S1: the HPLC results. Table S1: protein accession, name, and multiple of difference of DEPs. [file 5241902.f1.zip › 5241902.f1/Fig S1 (1).pdf]
